# Association of glutathione S-transferase M1 and T1 polymorphisms and temperament

**DOI:** 10.22099/mbrc.2017.4074

**Published:** 2017-09

**Authors:** Zahra Zendehboodi

**Affiliations:** Department of Biology, College of Sciences, Shiraz University, Shiraz, Iran

**Keywords:** Mizaj, Temperament, *GSTM1*, *GSTT1*, Polymorphism

## Abstract

Mizaj (Temperament) is one of the basic concepts of Iranian Traditional Medicine (ITM), which has great importance in diagnosis and treatment of diseases, as well as maintaining the ideal healthy state of an individual. However, very little is known about the biological mechanisms of mizaj dependence treatment in practical ITM. This study was aimed to evaluate any association between the mizaj and glutathione S-transferase M1 (*GSTM1*) and T1 (*GSTT1*) polymorphisms in healthy people. The samples included 247 healthy males from Fars province, southern Iran. The mizaj was determined using a self-reported mizaj identification scale. Subjects with equilibrium or any of four simple mizajes (warm, cold, moist, and dry) were included in the study. The *GSTM1* and *GSTT1* genotypes were determined using a polymerase chain reaction (PCR)-based method. No any differences in the frequency of *GSTM1*/*T1* polymorphism between equilibrium and each of other mizaj groups were found. However, when equilibrium, moist, and dry groups were pooled and as a "medium warmness" group were compared to the warm group, the frequency of *GSTT1*-null in the warm group was significantly higher compare to that in the medium warmness group. Also, the combination genotypes of *GSTM1* and *GSTT1* was associated with the warmness; that is, individuals with combination of *GSTM1* positive and *GSTT1* null were more susceptible to have a warm temperament. This study indicated that the mizaj could be affected by *GSTM1*/*T1* genes, although further research with larger samples is needed to reach full conclusions.

## INTRODUCTION

Iranian Traditional Medicine (ITM) which is one of the oldest schools of traditional medicine makes effort to provide the best possible ways for a person to have an optimum healthy life with minimal illness [1, 2]. One of the most important concepts of ITM is* "Mizaj"*. Mizaj (temperament) is a dominant quality which is a consequence of the interaction of elements with different qualities (Hot, Cold, Wet, Dry) in the human body. Mizaj is believed to have an important role in maintaining the ideal healthy state of an individual through affecting the normal physical and emotional characteristics and also the physiological functions of the body. Based on the degrees of warmness and wetness, the experts of ITM categorize the mizaj into nine major groups. These groups consist of one equilibrium mizaj which is in equilibrium for both warmness and wetness, four simple mizajes (warm, cold, moist, and dry) which are in equilibrium for one quality and out of that for another quality, and four combined mizajes (warm and moist, warm and dry, cold and moist, cold and dry) which are out of equilibrium for both qualities. According to ITM, a person is considered to be in a healthy state when his or her mizaj keeps its balance, and most of the diseases occur when the mizaj is disturbed [3, 4].

Glutathione transferases (GSTs) play a significant role in detoxification of a wide range of epoxides and certain other agents including environmental pollutants, chemotherapeutic agents, or dietary components. In humans, several classes of cytosolic GSTs were identified including theta (T), mu (M), and pi (P). *GSTT* and *GSTM* class genes have been localized to chromosome 22 and 1 respectively. Two major polymorphisms of the *GSTM1* (a member of class mu; OMIM: 138350) and *GSTT1 *(a member of class theta; OMIM: 600436) genes, were identified, which both result from gene deletion, and are associated with the absent of enzymatic activity. Homozygotes for the deleted gene are termed *GSTM1*/*GSTT1* null (*GSTM1*/*GSTT1-0*). Because of their role in detoxification, and their ability to modulate inflammatory processes, the potential for polymorphisms in GST to modulate disease has been the subject of considerable molecular epidemiologic studies [5].

In spite of the usefulness, the existing knowledge on the functional mechanisms of ITM based on temperament is not very extensive, and despite the progression of various sciences, the therapeutic methods of ITM are mainly based on practical experiences and texts belonging to a very far distant past. It is hope that new findings can help us to clarify the biological mechanisms of practical traditional medicine which in turn can be used in conjunction with and as an aid to the modern medicine. Phenotypic traits such as morphological, physiological and psychological features of one person are affected by the mizaj [3]. Considering the fact that the phenotypic variation can be partially attributed to genetic difference, identification of responsible genes of these temperaments could be helpful to explain the mechanism of these differences. To this aim, the present study has been carried out to assess any association between the mizaj and *GSTM1*/*T1* polymorphisms in healthy people.

## MATERIALS AND METHODS

**Study population: **The study was carried out on 247 mail healthy for blood donation, and they were aged between 20-40 years. All participants were from Fars province, southern Iran. Participants**’ **temperaments were determined using a self-reported mizaj identification scale constructed previously [6]. Subjects with equilibrium or any of four simple mizajes (warm, cold, moist, and dry) were included in the study. This study was approved by the Shiraz University ethics committee and each subject was informed about the objectives of the study. 

**DNA extraction and genotyping analysis: **Blood samples were obtained from healthy blood donors. Immediately after collection, whole blood was stored at -20°C until use. Genomic DNA for polymerase chain reaction (PCR) was isolated from whole blood using thawed blood samples by standard procedure [7]. *GSTM1 *and *GSTT1 *genotyping were determined by multiplex PCR, using β-globin gene as control, as previously described [8]. The PCR conditions for determining the *GSTM1* and *GSTT1* genotypes were the same as that reported previously [9-11]. The absence of amplified product was consistent with the homozygous null genotypes of *GSTM1* and *GSTT1*. Successful amplification by β-globin specific primers confirmed the proper function of the PCR reaction. Evaluating the polymorphism was the same as that reported previously [9-11].

**Statistical analysis: **Data were expressed as number of genotypes. The χ^2^ test was used to examine if the frequency of *GSTT1* and *GSTM1* polymorphisms in each mizaj group was differ from that in the equilibrium group. Logistic regression was used to calculate ORs and 95% CI for the association of temperament with the combination of *GSTT1* and *GSTM1* polymorphisms. The reference group consisted of individuals who had two active genotypes. Signiﬁcance was accepted at P<0.05. Statistical analysis was performed using the SPSS version 22.

## RESULTS

The distribution pattern of *GSTT1* and *GSTM1* polymorphisms in temperament groups were examined using χ^2^ test. According to table 1, the frequency of *GSTT1* and *GSTM1* polymorphisms in equilibrium group was not significantly differ from that in each of warm, moist, cold, and dry groups. However, it should be noted that the difference in the frequency of *GSTT1* polymorphism between equilibrium and warm groups was of borderline significance (χ^2^ = 3.12, *P* = 0.064); that is, compare to the equilibrium group, individuals with warm mizaj have a higher frequency of *GSTT1*-null. To examine more if warmness affected by *GSTT1* polymorphism, equilibrium, moist, and dry groups which all are in equilibrium for the warmness, were pooled and as a "medium warmness" group were compared to the warm group.

**Table 1:** Association between temperaments and polymorphisms of *GSTM1* and *GSTT1*

**Mizaj** **Groups**	***GSTM1***			**χ** ^2^	***P*** **-value**	***GSTT1***			**χ** ^2^	***P*** **-value**
	Active	Null	Total			Active	Null	Total		
**Equilibrium**	26	29	55			49	6	55		
**Warm**	25	30	55	0.037	1.00	42	13	55	3.12	0.064
**Cold**	17	15	32	0.277	0.660	26	6	32	1.05	0.345
**Moist**	24	29	53	0.043	0.849	46	7	53	0.135	0.774
**Dry**	21	31	52	0.515	0.560	46	6	52	0.011	1.00

*P*-value is represented for the χ^2^ between equilibrium group and each of four other groups

 As the table 2 showed, the frequency of *GSTT1*-null in the warm group was significantly higher compare to that in the medium warmness group (χ^2^ = 4.47, *P* = 0.047). On the other hand, harboring the null genotype of *GSTT1* lead to increase the warmness. The effect of *GSTM1* also examined in the same way, and no correlation was detected (Table 2). Then, was to investigate whether one null genotype could be compensated by an active genotype for the other GST isoenzymes, the association between combinations of the genotypes and temperament was examined. The reference group consisted of individuals who had two active genotypes. Because of small sample size, applying of this analysis between equilibrium group and each of four other mizaj groups was ignored and it was performed only between warm and medium warmness group. According to Table 3, compare to the combination of both *GSTM1* and *GSTT1* positive, individuals with combination of *GSTM1* positive and *GSTT1* null are more susceptible to have a warm temperament (OR = 2.936, 95% CI: 1.05-8.24, *P* = 0.041).

**Table 2:** Association between polymorphisms of *GSTM1* and *GSTT1* and warm temperament

**Mizaj** **Groups**	***GSTM1***			**χ** ^2^	***P*** **-value**	***GSTT1***			**χ** ^2^	***P*** **-value**
	Active	Null	Total			Active	Null	Total		
**Medium warmness**	71	89	160	0.019	1.00	141	19	160	4.47	0.047
**Warm**	25	30	55			42	13	55		

**Table3:** Association between combination of *GSTM1* and *GSTT1* polymorphisms and warm temperament

**Combination of genotypes**		**Medium warmness**	**Warm**	**OR**	**95% CI**	***P*** **-value**
*GSTM1 *	*GSTT1*					
Active	Active	61	17			
Null	Active	79	24	1.090	0.54-2.21	0.811
Active	Null	11	9	2.936	1.05-8.24	0.041
Null	Null	9	5	1.99	0.59-6.74	0.267

## Discussion

Mizaj is one of the basic concept of ITM and it plays an important role in diagnosis and treatment of diseases, as well as maintaining the optimum healthy state of an individual [3]. Recent studies indicate the association of temperament with autonomic and immune systems, blood groups, enzymatic variation, body composition and other biological factors [12-15]. Tao Ma’s research group examined gene expression information of cold syndrome using the microarray and systems biology methods and indicated that the genes related to cold syndrome involve in energy metabolism, which are correlated with the genes of neurotransmitters, hormones and cytokines in the neuro-endocrine-immune interaction network [16]. Using proteomic tools and network analysis on the mitochondria, it has been shown that there is a difference in the expression of some proteins and also protein-protein interaction networks of cold-dry temperament compare to that of Hot-wet ones [17]. Examining gene expression profile in CD4^+^ T cells of rheumatoid arthritis patients with cold or hat patterns showed that the genes involved in small G protein signaling pathways, oxidation–reduction in fatty acid metabolism and T cell proliferation were differentially expressed in these two temperaments [18]. This study was conducted to explore any relationship between the temperament and *GSTM1*/*T1* polymorphism. To this end, the frequency of *GSTM1*/*T1* polymorphism in equilibrium group were compared with that of other four simple mizaj groups. As the result showed, no any differences in the frequency of *GSTM1*/*T1* polymorphism between equilibrium group and each of cold, moist, and dry groups were found. However there was a borderline significantly increased *GSTT1*-nll in the warm group compared with that in the equilibrium group, and moreover when warm group compared to the medium warmness group, this difference achieve significance. However it should be mentioned that *GSTT1* null genotype affect the warmness when it is combined with *GSTM1* positive genotype (Table 3). On the other hand, the temperament of individuals harboring null genotype of *GSTT1* could be influenced by *GSTM1* polymorphism too. One limitation of this study which should be taken in to consideration is the missing health status of participants. However, despite some diseases are ignored in screening, it should be mentioned that usually most of volunteers verified for blood donation are in physical and mental health. In conclusion, this study is in agreement with other evidence which specify temperaments by distinct biological phenomena as it is support the view that the mizaj is affected by *GSTM1*/*T1* genes. This is a preliminary study and the first of its kind to be carried out, and therefore, further research is needed to reach full conclusions about the possible effect of these polymorphisms on the temperament.
